# Crystal structure of (*E*)-2-hy­droxy-1,2-di­phenyl­ethan-1-one oxime

**DOI:** 10.1107/S2056989017008866

**Published:** 2017-06-20

**Authors:** Hans Reuter, Coco K. Y. A. Okio

**Affiliations:** a Institute of Chemistry of New Materials, University of Osnabrück, Barbarastrasse 7, 49069 Osnabrück, Germany; b Departamento de Química, Facultad de Ciencias, Universidad Nacional de Colombia, Carerra 30 No 45-03, Bogotá, Colombia

**Keywords:** crystal structure, α-benzoinoxime, disorder

## Abstract

The title compound, C_14_H_13_NO_2_, is a commercially available material and can be used as a multidentate ligand. The mol­ecule of the asymmetric unit has an *R* configuration, while the corresponding *S*-configured mol­ecule of the racemic mixture is generated by a crystallographic centre of symmetry. Both hy­droxy groups (the H atom of the oxime group is equally disordered over two positions) are involved in hydrogen bonding, leading to the formation of chains extending parallel to [001].

## Abstract 2

The H atom of the oxime moiety is equally disordered over two positions, giving rise to two equivalent hydrogen bonds between adjacent mol­ecules.

## Chemical context   

The title compound (*E*)-2-hy­droxy-1,2-diphenyl-ethan-1-one oxime, C_14_H_13_NO_2_, is commercially available and can be used as a multidentate ligand for which many trivial names such as *cuprone* or *alpha-benzoin*, and abbreviations including AboH_2_, BzoxH_2_, are in use. Used for a long time for the determination of manganese or copper in steel (Feigl, 1923 ▸; Knowles, 1932 ▸; Kar, 1935 ▸), BzoxH_2_ has attracted considerable attention nowadays in the coordination chemistry of transition metals for the preparation of mol­ecular wheels and high-nuclearity metal units with copper, manganese or nickel cations (Stamatatos *et al.*, 2012 ▸; Vlahopoulou *et al.*, 2009 ▸; Koumousi *et al.* 2010 ▸; Karotsis *et al.*, 2009 ▸). In the course of a project to evaluate the reactivity of BzoxH_2_ towards organotin(IV) compounds, we obtained high-quality single crystals of the title compound which we have used for structure determination by X-ray diffraction.
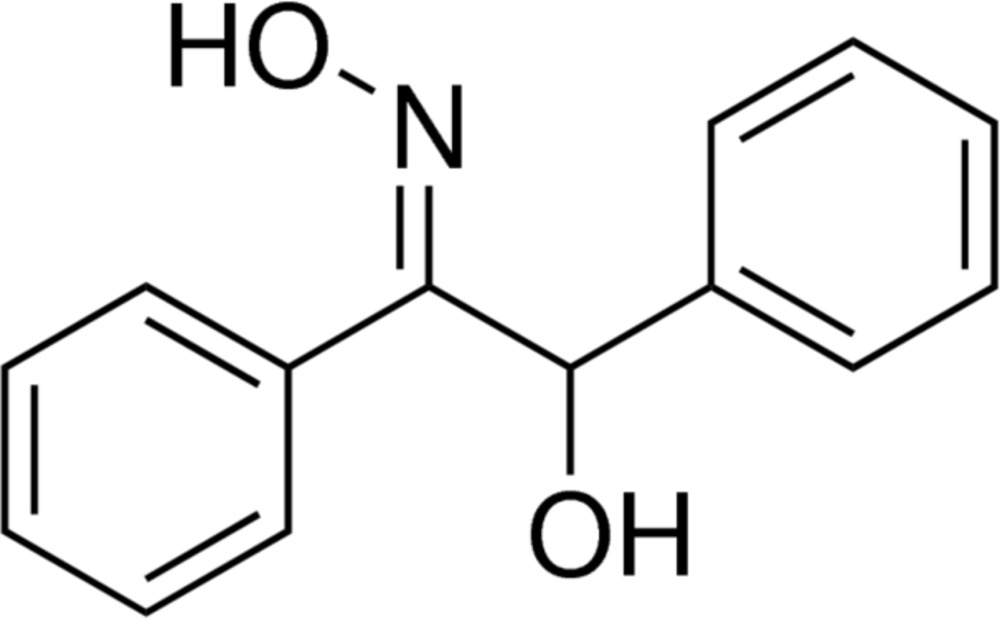


## Structural commentary   

**Figure 1:**
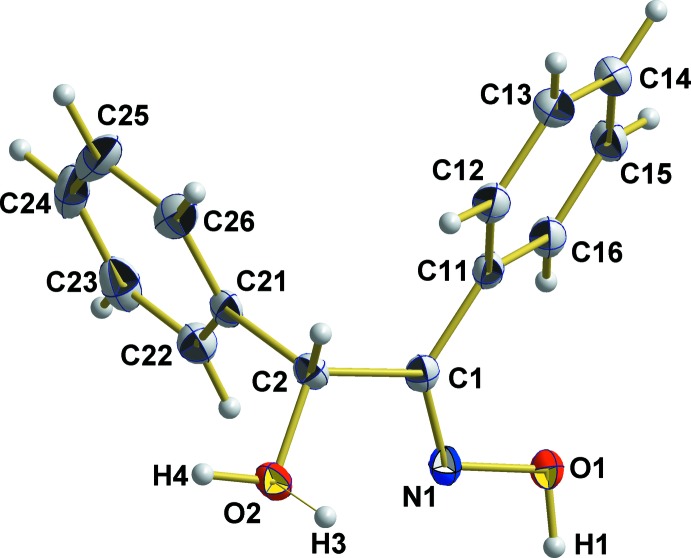
The asymmetric unit of the title compound, showing the atom-labelling scheme and displacement ellipsoids for the non-H atoms at the 50% probability level; split positions of the H atom attached to atom O2 are labelled H3 and H4.

BzoxH2 crystallizes in the centrosymmetric monoclinic space group *C*2/*c* with eight mol­ecules in the unit cell and one mol­ecule in the asymmetric unit. As the compound possesses an asymmetric carbon atom (C2), the mol­ecule of the asymmetric unit has an *R*-configuration while the corresponding *S*-configured mol­ecule of the racemic mixture is generated by a crystallographic centre of symmetry. Both mol­ecules also show the *E* configuration at the N=C double bond of the oxime moiety (Fig. 1 ▸).

The length [1.278 (2) Å] of the N=C double bond (Table 1 ▸) is consistent with the value of 1.281 (13) Å found in other oxime moieties (Allen *et al.*, 1987 ▸). In addition, this moiety is characterized by a bond angle of 115.5 (1)° at the N atom and of 102.1° at the O atom. The central C—C bond of the mol­ecule has a length of 1.525 (2), which is also in good accord­ance with a typical single bond between *sp* ^3^ (C2) and *sp* ^2^ (C1) hybridized C atoms. As a consequence of the different hybridization states, however, the bonds of these two carbon atoms to their phenyl groups are slightly different: 1.512 (2) Å for C2 and 1.484 (2) Å for C1, respectively. The hy­droxy group attached to C2 shows a C—O bond length of 1.425 (2) Å, which also lies in the normal range (1.421–1.433 Å) of a C_2_–CH–OH group (Allen *et al.*, 1987 ▸).

The two phenyl groups exhibit a mean C—C bond length of 1.387 (5) Å [variation: 1.374 (3)–1.398 (2) Å], in excellent agreement with the literature value (Allen *et al.*, 1987 ▸) of 1.387 (10) Å for C_ar_—C_ar_. The mean value of the endocyclic bond angles within the phenyl rings is 120.0 (5)° with minima at the *ipso* carbon atoms C11 [118.3 (1)°] and C21 [119.1 (1)°]. The phenyl rings form an inter­planar angle of 80.72 (5)°.

## Supra­molecular features   

**Figure 2:**
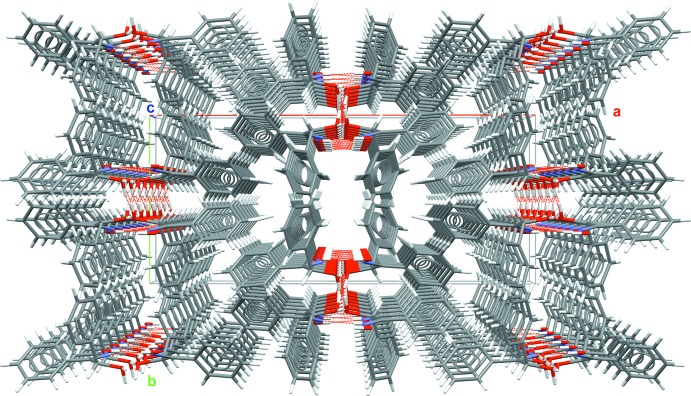
Crystal packing showing the tube-like arrangement of the mol­ecules along [001].

**Figure 3:**
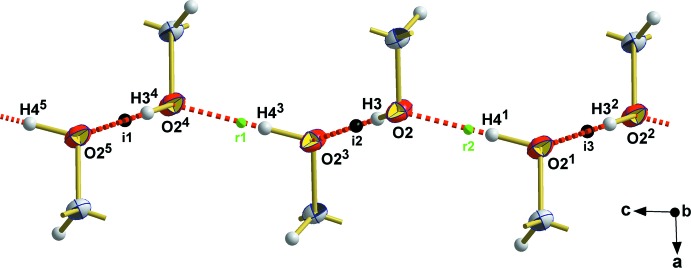
Detail of the one-dimensional hydrogen-bonding system (red dashed lines) derived from the hy­droxy group attached to the C atom looking down [010]; displacement ellipsoids for the non-H atoms are drawn at the 50% probability level. Groups attached to C atoms have been omitted for clarity. Small black dots visualize the position of an inversion center [i1: 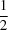, 0, 1; i2: 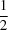, 0, 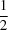; i3: 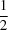, 0, 0], green dots the position of twofold rotation axes [r1: 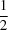, *y*, 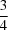; r2: 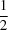, *y*, 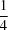]. [Symmetry codes used to generate equivalent atoms: (1) 1 − *x*, *y*, 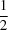 − *z*; (2) *x*, −*y*, −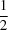 + *z*; (3) 1 − *x*, −*y*, 1 − *z*; (4) *x*, −*y*, 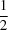 + *z*; (5) 1 − *x*, *y*, 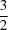 − *z*.]

**Figure 4:**
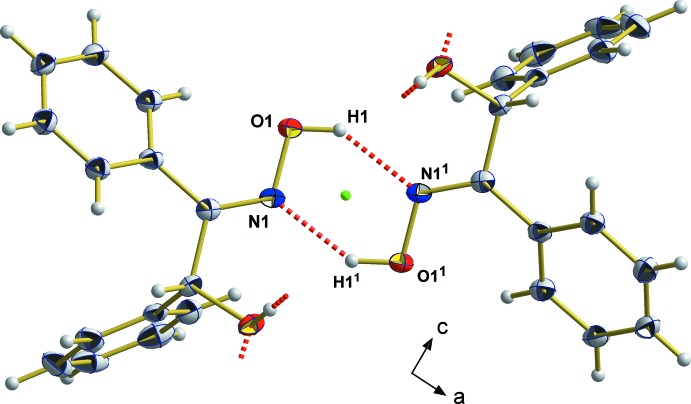
Hydrogen-bonding system (red dashed lines) between the oxime groups of two neighbouring mol­ecules looking down [010]; displacement ellipsoids for the non-H atoms are given at the 50% probability level. The small green dot visualizes the position of the twofold rotation axis at 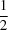, *y*, 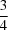. [Symmetry codes used to generate equivalent atoms: (1) 1 − *x*, *y*, 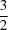 − *z*.]

The mol­ecule possesses two hy­droxy groups which, in principle, can act as donors and acceptors for hydrogen bonding while the N atom of the oxime moiety can only act as an acceptor atom in the formation of hydrogen bonds. In fact, the crystal packing (Fig. 2 ▸) with its clear separation of polar and non-polar moieties, results from two different types of hydrogen bonds (Table 2 ▸), giving rise to a one-dimensional tube-like arrangement of the mol­ecules propagating along [001]. In the first type of hydrogen bond, only the hy­droxy group attached to the carbon atom C2 is involved, acting both as hydrogen-donor and hydrogen-acceptor groups (Fig. 3 ▸). Since the oxygen atoms of the resulting hydrogen bonds are related to each other by a centre of symmetry [O2⋯O2^ii^ = 2.829 (2) Å, 〈O2—H3⋯O2^ii^ = 164°; symmetry code: (ii) = −*x* + 1, −*y*, −*z* + 1] and a twofold rotation axis [O2⋯O2^iii^ = 2.806 (2) Å, 〈O2—H4⋯O1^iii^ = 175°; symmetry code (iii) = −*x* + 1, *y*, −*z* + 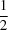], respectively, the hydrogen atom of the hy­droxy group breaks space-group symmetry, which was considered in the structure model by two equally disordered split positions [H3/H4] of this hydrogen atom. While this kind of hydrogen-bonding system extends to an infinite number of mol­ecules, the second type of hydrogen bond is limited to two neigbouring mol­ecules. It involves the hy­droxy group of the oxime moiety that acts as an H-atom donor forming mutual hydrogen bonds with the nitro­gen atom of the oxime moiety of a neighbouring mol­ecule, giving rise to two equivalent hydrogen bonds [O1⋯N1^i^ = 2.805 (2) Å, 〈O1—H1⋯ N1^i^ = 144°; symmetry code: (i) = −*x* + 1, *y*, −*z* + 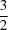] between these two mol­ecules (Fig. 4 ▸). The two mol­ecules within the resulting six-membered ring are related to each other by a twofold rotation axis.

## Synthesis and crystallization   

In a typical experiment, *α*-benzoinoxime was refluxed with di-*n*-butyl­tin oxide, C_8_H_18_OSn, in ethanol for 2.5 h. Single crystals of the title compound suitable for X-ray diffraction were obtained from the ethano­lic solution layered with *n*-hexane.

## Refinement details   

Crystal data, data collection and structure refinement details are summarized in Table 3 ▸. All H atoms were clearly identified in difference Fourier syntheses. Those of the carbon skeleton were calculated assuming idealized geometries and allowed to ride on the carbon atoms with 1.00 Å for *sp* ^3^-hybridized and 0.95 Å for aromatic H atoms, and with *U*_iso_(H) = 1.2*U*_eq_(C). The H atoms of the two hy­droxy groups were modelled with a common O—H distance of 0.96 Å before they were fixed and allowed to ride on the corresponding oxygen atom with *U*_iso_(H) = 1.2*U*_eq_(O). Disorder of the hy­droxy group attached to C2 was taken into account reducing the site occupancy of both H atoms to one-half. This suggestion was confirmed by difference-Fourier maps that clearly showed both positions.

## Supplementary Material

Crystal structure: contains datablock(s) I. DOI: 10.1107/S2056989017008866/wm5397sup1.cif

Structure factors: contains datablock(s) I. DOI: 10.1107/S2056989017008866/wm5397Isup2.hkl

Supporting information file. DOI: 10.1107/S2056989017008866/wm5397Isup3.cml

CCDC reference: 1556039

Additional supporting information:  crystallographic information; 3D view; checkCIF report

## Figures and Tables

**Table 1:** Selected geometric parameters (Å, °)

O1—N1	1.404 (1)	C1—C2	1.525 (2)
C1—N1	1.278 (2)	O2—C2	1.425 (2)
			
N1—C1—C2	114.3 (1)	C1—N1—O1	115.5 (1)
C11—C1—C2	117.7 (1)	O2—C2—C1	110.1 (1)

**Table 2:** Hydrogen-bond geometry (Å, °)

*D*—H⋯*A*	*D*—H	H⋯*A*	*D*⋯*A*	*D*—H⋯*A*
O1—H1⋯N1^i^	0.96	1.97	2.805 (2)	144
O2—H3⋯O2^ii^	0.96	1.89	2.829 (2)	164
O2—H4⋯O2^iii^	0.96	1.85	2.806 (2)	175

Symmetry codes: (i) 
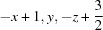
; (ii) 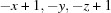; (iii) 
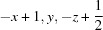
.

**Table 3:** Experimental details

Crystal data	
Chemical formula	C_14_H_13_NO_2_
*M*_r_	227.25
Crystal system, space group	Monoclinic, *C*2/*c*
Temperature (K)	100
*a*, *b*, *c* (Å)	24.1434 (9), 10.5348 (4), 8.9006 (4)
β (°)	93.042 (2)
*V* (Å^3^)	2260.64 (16)
*Z*	8
Radiation type	Mo *K*α
μ (mm^−1^)	0.09
Crystal size (mm)	0.37 × 0.32 × 0.11

Data collection	
Diffractometer	Bruker APEXII CCD
Absorption correction	Multi-scan (*SADABS*; Bruker, 2009 ▸)
*T*_min_, *T*_max_	0.968, 0.990
No. of measured, independent and observed [*I* > 2σ(*I*)] reflections	50071, 2005, 1765
*R*_int_	0.043
(sin θ/λ)_max_ (Å^−1^)	0.595

Refinement	
*R*[*F* ^2^ > 2σ(*F* ^2^)], *wR*(*F* ^2^), *S*	0.036, 0.088, 1.08
No. of reflections	2005
No. of parameters	157
H-atom treatment	H-atom parameters constrained
Δρ_max_, Δρ_min_ (e Å^−3^)	0.22, −0.18

Computer programs: *APEX2* (Bruker, 2009 ▸), *SAINT* (Bruker, 2009 ▸), *SHELXS97* (Sheldrick, 2008 ▸), *SHELXL2014* (Sheldrick, 2015 ▸), *DIAMOND* (Brandenburg, 2006 ▸) and *Mercury* (Macrae *et al.*, 2008 ▸).

## supplementary crystallographic information

### Crystal data

**Table:**

C_14_H_13_NO_2_	*F*(000) = 960
*M**_r_* = 227.25	*D*_x_ = 1.335 Mg m^−^^3^
Monoclinic, *C*2/*c*	Mo *K*α radiation, λ = 0.71073 Å
*a* = 24.1434 (9) Å	Cell parameters from 1932 reflections
*b* = 10.5348 (4) Å	θ = 3.1–24.4°
*c* = 8.9006 (4) Å	µ = 0.09 mm^−^^1^
β = 93.042 (2)°	*T* = 100 K
*V* = 2260.64 (16) Å^3^	Block, colourless
*Z* = 8	0.37 × 0.32 × 0.11 mm

### Data collection

**Table:**

Bruker APEXII CCD diffractometer	1765 reflections with *I* > 2σ(*I*)
φ and ω scans	*R*_int_ = 0.043
Absorption correction: multi-scan (*SADABS*; Bruker, 2009)	θ_max_ = 25.0°, θ_min_ = 2.1°
*T*_min_ = 0.968, *T*_max_ = 0.990	*h* = −28→28
50071 measured reflections	*k* = −12→12
2005 independent reflections	*l* = −10→10

### Refinement

**Table:**

Refinement on *F*^2^	0 restraints
Least-squares matrix: full	Hydrogen site location: mixed
*R*[*F*^2^ > 2σ(*F*^2^)] = 0.036	H-atom parameters constrained
*wR*(*F*^2^) = 0.088	*w* = 1/[σ^2^(*F*_o_^2^) + (0.0361*P*)^2^ + 2.1083*P*] where *P* = (*F*_o_^2^ + 2*F*_c_^2^)/3
*S* = 1.08	(Δ/σ)_max_ < 0.001
2005 reflections	Δρ_max_ = 0.22 e Å^−^^3^
157 parameters	Δρ_min_ = −0.18 e Å^−^^3^

### Special details

**Table:**

Geometry. All esds (except the esd in the dihedral angle between two l.s. planes) are estimated using the full covariance matrix. The cell esds are taken into account individually in the estimation of esds in distances, angles and torsion angles; correlations between esds in cell parameters are only used when they are defined by crystal symmetry. An approximate (isotropic) treatment of cell esds is used for estimating esds involving l.s. planes.

### Fractional atomic coordinates and isotropic or equivalent isotropic displacement parameters (Å^2^)

**Table:**

	*x*	*y*	*z*	*U*_iso_*/*U*_eq_	Occ. (<1)
O1	0.43413 (4)	0.17890 (9)	0.80605 (10)	0.0241 (2)	
H1	0.4693	0.1776	0.8619	0.029 (3)*	
C1	0.41037 (6)	0.14256 (12)	0.55975 (15)	0.0203 (3)	
N1	0.44993 (5)	0.16404 (11)	0.65751 (12)	0.0214 (3)	
O2	0.48687 (4)	0.10235 (9)	0.40164 (11)	0.0252 (3)	
H3	0.4938	0.0231	0.4524	0.029 (3)*	0.5
H4	0.4959	0.0973	0.2981	0.029 (3)*	0.5
C2	0.42878 (6)	0.12695 (13)	0.39955 (15)	0.0217 (3)	
H2	0.4086	0.0531	0.3519	0.029 (3)*	
C11	0.35015 (6)	0.13354 (13)	0.58375 (15)	0.0210 (3)	
C12	0.31705 (6)	0.04617 (14)	0.50248 (16)	0.0253 (3)	
H12	0.3332	−0.0072	0.4307	0.0306 (19)*	
C13	0.26100 (6)	0.03627 (15)	0.52511 (17)	0.0302 (4)	
H13	0.2391	−0.0245	0.4700	0.0306 (19)*	
C14	0.23671 (6)	0.11432 (16)	0.62737 (17)	0.0311 (4)	
H14	0.1982	0.1071	0.6432	0.0306 (19)*	
C15	0.26863 (6)	0.20283 (15)	0.70637 (17)	0.0283 (3)	
H15	0.2518	0.2576	0.7755	0.0306 (19)*	
C16	0.32497 (6)	0.21281 (14)	0.68598 (16)	0.0247 (3)	
H16	0.3466	0.2738	0.7417	0.0306 (19)*	
C21	0.41529 (5)	0.24451 (14)	0.30732 (15)	0.0223 (3)	
C22	0.43786 (6)	0.36078 (15)	0.34820 (17)	0.0292 (3)	
H22	0.4627	0.3671	0.4344	0.040 (2)*	
C23	0.42453 (7)	0.46786 (16)	0.2644 (2)	0.0373 (4)	
H23	0.4397	0.5478	0.2940	0.040 (2)*	
C24	0.38915 (7)	0.45886 (17)	0.13770 (19)	0.0402 (4)	
H24	0.3797	0.5327	0.0807	0.040 (2)*	
C25	0.36771 (7)	0.34293 (18)	0.09425 (18)	0.0404 (4)	
H25	0.3440	0.3362	0.0059	0.040 (2)*	
C26	0.38058 (6)	0.23623 (16)	0.17912 (17)	0.0312 (4)	
H26	0.3654	0.1564	0.1491	0.040 (2)*	

### Atomic displacement parameters (Å^2^)

**Table:**

	*U*^11^	*U*^22^	*U*^33^	*U*^12^	*U*^13^	*U*^23^
O1	0.0247 (5)	0.0303 (6)	0.0176 (5)	0.0021 (4)	0.0046 (4)	−0.0010 (4)
C1	0.0231 (7)	0.0159 (7)	0.0221 (7)	0.0033 (5)	0.0038 (5)	0.0023 (5)
N1	0.0240 (6)	0.0217 (6)	0.0189 (6)	0.0021 (5)	0.0057 (5)	−0.0002 (5)
O2	0.0211 (5)	0.0259 (5)	0.0292 (5)	0.0051 (4)	0.0078 (4)	0.0033 (4)
C2	0.0185 (7)	0.0234 (7)	0.0236 (7)	0.0017 (5)	0.0041 (5)	−0.0008 (6)
C11	0.0230 (7)	0.0209 (7)	0.0194 (7)	0.0025 (5)	0.0037 (5)	0.0051 (5)
C12	0.0273 (8)	0.0258 (8)	0.0230 (7)	0.0027 (6)	0.0029 (6)	0.0014 (6)
C13	0.0256 (8)	0.0347 (9)	0.0301 (8)	−0.0045 (6)	−0.0001 (6)	0.0031 (7)
C14	0.0224 (8)	0.0421 (9)	0.0292 (8)	0.0007 (7)	0.0052 (6)	0.0090 (7)
C15	0.0266 (8)	0.0332 (8)	0.0259 (8)	0.0060 (6)	0.0084 (6)	0.0035 (6)
C16	0.0268 (8)	0.0245 (7)	0.0232 (7)	0.0024 (6)	0.0043 (6)	0.0022 (6)
C21	0.0209 (7)	0.0265 (8)	0.0203 (7)	0.0057 (6)	0.0081 (5)	0.0002 (6)
C22	0.0269 (8)	0.0314 (8)	0.0297 (8)	0.0012 (6)	0.0051 (6)	0.0027 (7)
C23	0.0397 (9)	0.0274 (9)	0.0465 (10)	0.0026 (7)	0.0177 (8)	0.0054 (7)
C24	0.0503 (10)	0.0395 (10)	0.0325 (9)	0.0225 (8)	0.0189 (8)	0.0163 (8)
C25	0.0478 (10)	0.0527 (11)	0.0206 (8)	0.0244 (9)	0.0007 (7)	0.0016 (7)
C26	0.0346 (8)	0.0353 (9)	0.0239 (8)	0.0095 (7)	0.0028 (6)	−0.0052 (7)

### Geometric parameters (Å, º)

**Table:**

O1—N1	1.404 (1)	C14—C15	1.378 (2)
O1—H1	0.9600	C14—H14	0.9500
C1—N1	1.278 (2)	C15—C16	1.386 (2)
C1—C11	1.484 (2)	C15—H15	0.9500
C1—C2	1.525 (2)	C16—H16	0.9500
O2—C2	1.425 (2)	C21—C22	1.381 (2)
O2—H3	0.9600	C21—C26	1.382 (2)
O2—H4	0.9600	C22—C23	1.381 (2)
C2—C21	1.512 (2)	C22—H22	0.9500
C2—H2	1.0000	C23—C24	1.382 (3)
C11—C12	1.396 (2)	C23—H23	0.9500
C11—C16	1.398 (2)	C24—C25	1.374 (3)
C12—C13	1.383 (2)	C24—H24	0.9500
C12—H12	0.9500	C25—C26	1.380 (2)
C13—C14	1.380 (2)	C25—H25	0.9500
C13—H13	0.9500	C26—H26	0.9500
			
N1—O1—H1	102.1	C13—C14—H14	120.2
N1—C1—C11	128.03 (12)	C14—C15—C16	120.70 (14)
N1—C1—C2	114.3 (1)	C14—C15—H15	119.6
C11—C1—C2	117.7 (1)	C16—C15—H15	119.6
C1—N1—O1	115.5 (1)	C15—C16—C11	120.25 (14)
C2—O2—H3	108.1	C15—C16—H16	119.9
C2—O2—H4	105.7	C11—C16—H16	119.9
H3—O2—H4	111.2	C22—C21—C26	119.12 (14)
O2—C2—C21	109.84 (11)	C22—C21—C2	120.85 (13)
O2—C2—C1	110.1 (1)	C26—C21—C2	120.02 (13)
C21—C2—C1	110.75 (11)	C23—C22—C21	120.32 (15)
O2—C2—H2	108.7	C23—C22—H22	119.8
C21—C2—H2	108.7	C21—C22—H22	119.8
C1—C2—H2	108.7	C22—C23—C24	120.07 (16)
C12—C11—C16	118.33 (13)	C22—C23—H23	120.0
C12—C11—C1	120.41 (12)	C24—C23—H23	120.0
C16—C11—C1	121.26 (13)	C25—C24—C23	119.88 (15)
C13—C12—C11	120.81 (13)	C25—C24—H24	120.1
C13—C12—H12	119.6	C23—C24—H24	120.1
C11—C12—H12	119.6	C24—C25—C26	119.95 (16)
C14—C13—C12	120.31 (14)	C24—C25—H25	120.0
C14—C13—H13	119.8	C26—C25—H25	120.0
C12—C13—H13	119.8	C25—C26—C21	120.62 (16)
C15—C14—C13	119.58 (14)	C25—C26—H26	119.7
C15—C14—H14	120.2	C21—C26—H26	119.7
			
C11—C1—N1—O1	1.1 (2)	C14—C15—C16—C11	0.5 (2)
C2—C1—N1—O1	179.96 (10)	C12—C11—C16—C15	0.8 (2)
N1—C1—C2—O2	17.34 (16)	C1—C11—C16—C15	179.89 (13)
C11—C1—C2—O2	−163.64 (11)	O2—C2—C21—C22	−61.09 (16)
N1—C1—C2—C21	−104.31 (14)	C1—C2—C21—C22	60.68 (16)
C11—C1—C2—C21	74.72 (15)	O2—C2—C21—C26	118.01 (14)
N1—C1—C11—C12	−143.31 (15)	C1—C2—C21—C26	−120.23 (14)
C2—C1—C11—C12	37.82 (18)	C26—C21—C22—C23	1.9 (2)
N1—C1—C11—C16	37.6 (2)	C2—C21—C22—C23	−178.96 (13)
C2—C1—C11—C16	−141.28 (13)	C21—C22—C23—C24	−1.0 (2)
C16—C11—C12—C13	−1.5 (2)	C22—C23—C24—C25	−0.7 (2)
C1—C11—C12—C13	179.39 (13)	C23—C24—C25—C26	1.4 (2)
C11—C12—C13—C14	0.9 (2)	C24—C25—C26—C21	−0.5 (2)
C12—C13—C14—C15	0.3 (2)	C22—C21—C26—C25	−1.2 (2)
C13—C14—C15—C16	−1.1 (2)	C2—C21—C26—C25	179.69 (13)

### Hydrogen-bond geometry (Å, º)

**Table:**

*D*—H···*A*	*D*—H	H···*A*	*D*···*A*	*D*—H···*A*
O1—H1···N1^i^	0.96	1.97	2.805 (2)	144
O2—H3···O2^ii^	0.96	1.89	2.829 (2)	164
O2—H4···O2^iii^	0.96	1.85	2.806 (2)	175

Symmetry codes: (i) −*x*+1, *y*, −*z*+3/2; (ii) −*x*+1, −*y*, −*z*+1; (iii) −*x*+1, *y*, −*z*+1/2.
